# Data on macro(micro)plastics and hydrophobic organic contaminants in the Gulf of Guinea coastal psammitic beaches

**DOI:** 10.1016/j.dib.2022.108623

**Published:** 2022-09-20

**Authors:** Nsikak U. Benson, Omowunmi H. Fred-Ahmadu, Olusegun O. Ayejuyo

**Affiliations:** a Department of Chemistry^1^, Covenant University, Km 10 Idiroko Road, Ota, Nigeria; b Department of Chemistry, University of Lagos, Akoka, Nigeria

**Keywords:** Microplastics, Hydrophobic organic contaminants, Sediments, Contamination, Gulf of Guinea

## Abstract

The ubiquity of microplastics in coastal environments and marine ecosystems is a significant concern because they have a strong affinity for organic contaminants. This paper presents the first reported data on hydrophobic organic contaminants (HOCs) and microplastics particles (MPs, 1–5 mm) in lagoon and beach sediments along the Gulf of Guinea coastline (SE Atlantic). Sampling was carried out between August and November 2019. Ten sites were designated for each location, and sediment samples were taken along three transects: high waterline, drift waterline, and current waterline. Sediment samples were extracted through density floatation procedure and sieving. Primary data on polycyclic aromatic hydrocarbons (PAHs), polychlorinated biphenyls (PCBs), and organochlorine pesticides (OCPs) associated with MPs are provided, as well as detailed information on sampling coordinates, plastic types, and their relative abundance. Refer to the research publication ``Microplastics and associated organic pollutants in beach sediments from the Gulf of Guinea (SE Atlantic) coastal ecosystems'' (Fred-Ahmadu et al., 2022) for detailed discussion and interpretation of the reported data.

**Specifications Table**

**Table:** 

Subject	Environmental Chemistry, Marine Pollution
Specific subject area	Microplastic pollution
Type of data	Table Graph Figure
How the data were acquired	Hand-operated 0.5 × 0.5 ×0.2 quadrat; Agilent 7890A Gas Chromatography with Agilent 5975 Mass Spectroscopy Detector (GC–MS), Agilent 720-ES Inductively Coupled Plasma–Optical Emission Spectrometry (ICP–OES). Data was acquired and processed using Microsoft Office Excel 2016 and AddinSoft XLSTAT 2019. Analytical column was an MS C18 column (Agilent Technologies, Waldbronn, Germany) was used (450°C: 25 m × 320 μm × 0 μm particle size); Agilent 630 Cary ATR-FTIR with diamond crystal.
Data format	Raw Analysed
Description of data collection	Lagoon and beach sediment samples were collected. Map of study area and sampling locations. Microplastics (1–5 mm) distribution and abundance by size and colour. MPs were extracted and analysed for polycyclic aromatic hydrocarbons (PAHs), polychlorinated biphenyls (PCBs) and organochlorine pesticides (OCPs). Priority PAHs included naphthalene (NaP), 2-methyl naphthalene (2MNaP), acenaphthylene (ACNY), acenaphthene (ACN), fluorene (FLO), phenanthrene (Phen), fluoranthene (FLA), pyrene (PYR), benz[a]anthracene (BaA), benzo[k]fluoranthene (BkF), benzo[b]fluoranthene (BbF), dibenz[a,h]anthracene (DahA), and benzo[g,h,i]perylene (BghiP); 17 PCB congeners included PCBs 1, 5, 18, 31, 44, 52, 66, 86, 101, 110, 137, 141, 151, 170, 181, 187 and 206; and 14 OCPs, δ-lindane, α-lindane, β-lindane, γ-lindane, heptachlor, aldrin, heptachlor epoxide, endosulfan I, dichlorodiphenyl dichloro ethylene (p,p’- DDE), endrin, endosulfan II, dichloro diphenyl dichloro ethane (p,p’- DDD), dichlorodiphenyl trichloro ethane (p,p’- DDT) and methoxychlor were analysed.
Data source location	Lagos lagoon, Atican beach, Oniru beach, Elegushi beach, Eleko beach, Gulf of Guinea, Southeast Atlantic Ocean.
Data accessibility	Repository name: Mendeley Data Data identification number: N/A Direct URL to data: https://doi.org/10.17632/b9fb98hsmb.1
Related research article	O.H. Fred-Ahmadu, I.T. Tenebe, O.O. Ayejuyo, N.U. Benson (2022), Microplastics and associated organic pollutants in beach sediments from the Gulf of Guinea (SE Atlantic) coastal ecosystems, Chemosphere, 298, 134193, 10.1016/j.chemosphere.2022.134193

## Value of the Data

• First report on the occurrence and distribution of microplastics-sorbed PAHs, OCPs and PCBs in beach sediments along the Gulf of Guinea coast (SE Atlantic) is documented.
• Microplastics-HOCs contamination in lagoon and psammitic beach sediments is reported.
• A baseline data for future investigations on microplastic contamination, origin and impacts of organic contaminants in the studied coastlines is established.
• Data could be employed to conduct a comparative assessment of microplastic pollution in marine shoreline sediments from other regions of the world.
• The data will contribute towards addressing UN SDGs 14 and 15, and stakeholders’ campaign against plastic pollution.

## 1 Data Description

**Fig. 1:**
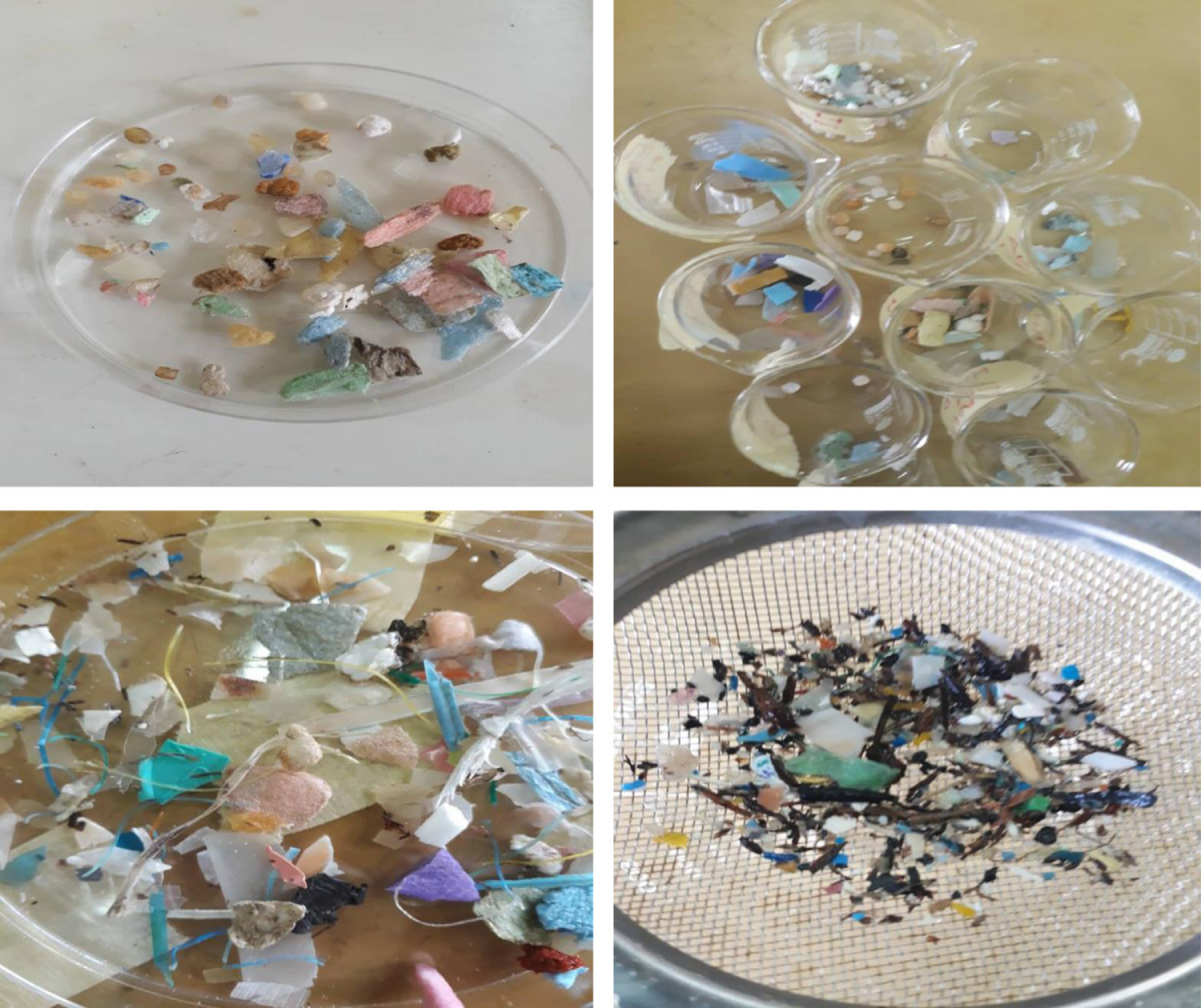
Samples of polymers collected.

**Fig. 2:**
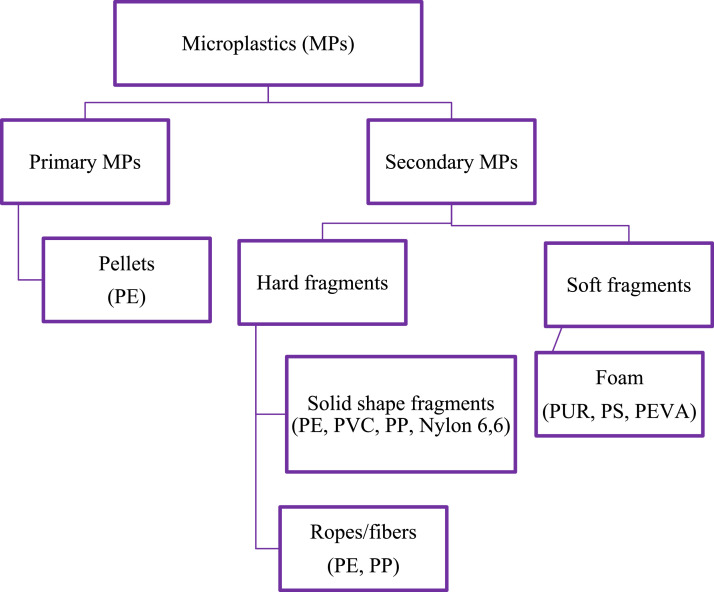
Physical classification of MPs.

**Fig. 3:**
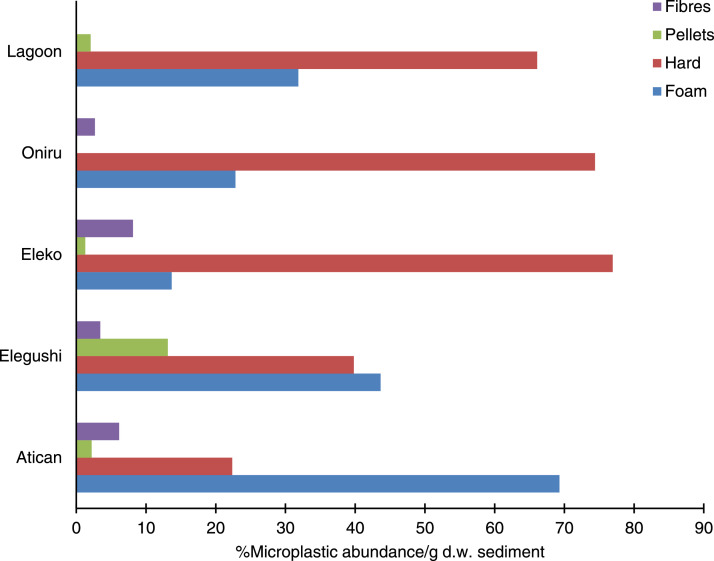
Relative abundance of polymer types.

**Fig. 4:**
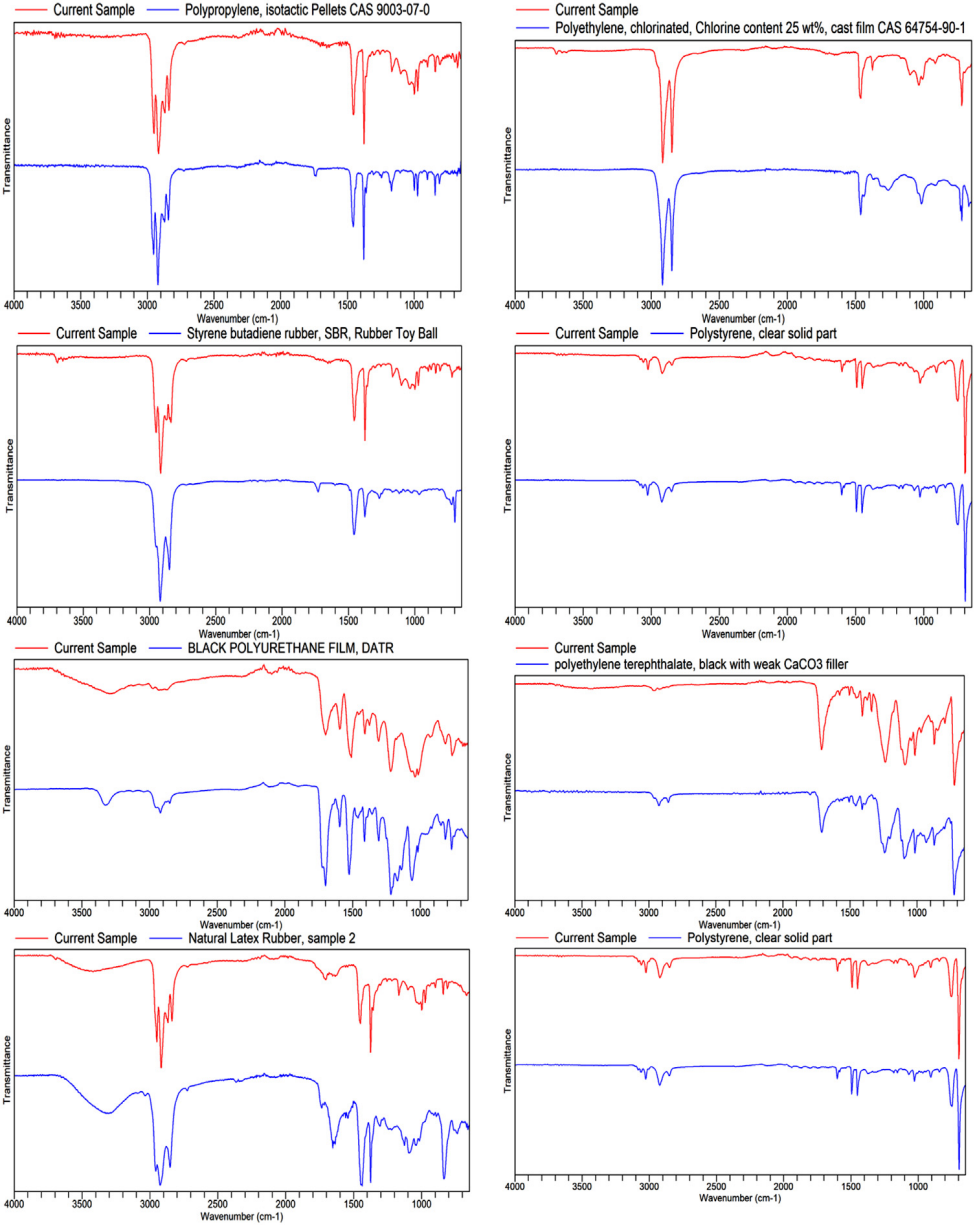
Spectra of sedimentary microplastics identified using FT-IR spectroscopy.

The dataset comprises a survey of microplastics (MPs, 1–5 mm) and concentrations of hydrophobic organic contaminants (PAHs, OCPs, PCBs) in psammitic sediment samples collected from designated sampling locations along the Lagos lagoon, Atican, Elegushi, Eleko, and Oniru beaches' coastlines in the Gulf of Guinea (SE Atlantic). Tables 1 and 2 provide the sampling codes and location descriptions of the study areas. Fig. 1 shows photographs of microplastics extracted from sediment samples, while Fig. 2 presents the physical characterisation of microplastics. The Attenuated Total Reflectance – Fourier Transform Infra-Red (ATR-FTIR) Spectrometry was used to determine the polymer types as presented in Table 3. The concentrations of hydrophobic organic contaminants detected in sedimentary microplastic samples collected along the beach and lagoon drift and high waterlines have been reported [1] (Figs. 3 and Fig. 3, Fig. 4).

**Table 1:** Sample codes and their descriptions for beach samples.

S/N	Sample code	Description
1	AHF	Atican beach High waterline Foam
2	ADF	Atican beach Drift waterline Foam
3	AHH	Atican beach High waterline Hard plastics
4	ADH	Atican beach Drift waterline Hard plastics
5	OHF	Oniru beach High waterline Foam
6	OHR	Oniru beach High waterline Fibre/ropes
7	ODH	Oniru beach Drift waterline hard plastics
8	ODF	Oniru beach Drift waterline foam
9	ODR	Oniru beach Drift waterline ropes/fibre
10	OHH	Oniru beach High waterline Hard plastics
11	EHH	Elegushi beach high waterline hard plastics
12	EHF	Elegushi beach high waterline foam
13	EDF	Elegushi beach Drift waterline foam
14	EHP	Elegushi beach High waterline pellets
15	EDH	Elegushi beach Drift waterline hard
16	EDP	Elegushi beach Drift waterline pellets
17	AHC	Composite High waterline sample from Atican
18	ADC	Composite Drift waterline sample from Atican
19	OHC	Composite High waterline sample from Oniru
20	ODC	Composite Drift waterline sample from Oniru
21	EHC	Composite High waterline sample from Elegushi
22	EDC	Composite Drift waterline sample from Elegushi
23	KHC	Composite High waterline sample from Eleko
24	KHH	High waterline hard plastics from Eleko
25	KHR	High waterline fibre from Eleko
26	KDH	Drift waterline hard plastics from Eleko
27	KDR	Drift waterline fibres from Eleko
28	0KDC	Composite Drift waterline sample from Eleko
29	KHF	High waterline foam sample from Eleko

**Table 2:** Sample codes and descriptions for Lagos lagoon samples.

S/N	Sample code	Description
1	NIH	Hard plastics from NIOMR
2	NIF	Foam samples from NIOMR
3	NIS	Slipper-like plastics from NIOMR
4	UNS	Slipper-like plastics from Unilag waterfront
5	UNH	Hard plastics from Unilag waterfront
6	UNF	Foam samples from Unilag waterfront
7	USH	Hard plastics from US Embassy
8	USF	Foam samples from US Embassy
9	USS	Slipper-like plastics from US Embassy
10	NIC	Composite sample from NIOMR
11	UNC	Composite sample from Unilag waterfront
12	USC	Composite sample from US Embassy
13	NIO	NIOMR sampling site
14	UNI	Unilag sampling site
15	USE	US Embassy sampling site

**Table 3:** Types of plastic (polymer) types identified for lagoon and beach locations.

Characteristic		Type of plastic	Atican	%	Elegushi	%	Oniru	%	Eleko	%	Lagoon	%	Total plastic type for study locations	%
Physical morphology of MPs		Foam	158	69.2	522	43.6	395	22.8	32	13.6	93	31.8	1200	32.6
		Hard	51	22.4	476	39.8	1287	74.4	180	76.9	193	66.1	2187	59.4
		Pellets	5	2.2	157	13.1	2	0.1	3	1.3	6	2.0	173	4.7
		Fibres	14	6.1	41	3.4	46	2.7	19	8.1	0	0	120	3.3
	Total		228		1196		1730		234		292		3680	

		Acronym												

Polymer types		PE	38	16.7	407	34.0	897	51.8	107	45.7	25	8.6	1474	40.1
		PP	22	9.6	208	17.4	413	23.9	56	23.9	23	7.9	722	19.6
		PS	123	53.9	491	41.0	322	18.6	23	9.8	53	18.1	1012	27.5
		PUR	19	8.3	21	1.8	57	3.3	9	3.8	20	6.8	126	3.4
		PEVA	9	3.9	10	0.8	16	0.9	0	0	28	9.6	63	1.7
		PET	0	0	4	0.3	2	0.1	3	1.3	127	43.5	136	3.6
		PA	8	3.5	40	3.3	33	1.9	22	9.4	9	3.1	112	3.0
		PVC	2	0.9	3	0.3	0	0	6	2.6	0	0	11	0.2
		Others	7	3.1	12	1.0	8	0.5	8	3.4	7	2.4	42	1.1
	Total		228		1196		1730		234		292		3680

## 2 Experimental Design, Materials and Methods

### 2.1 Sediment Sampling and Preservation

Sediment samples were collected between August and November, 2019 at 40 sites from five lagoon and beach locations namely Lagos lagoon, Atican, Elegushi, Eleko and Oniru along the coastlines of the Gulf of Guinea (SE Atlantic). The lagoon and beach locations were divided into three transects (High (HW), drift (DW), and current (CW) waterlines) and beach sediment samples were taken from 10 sites (100 m inter-site apart) per transect using a 0.5 × 0.5 × 0.2 m quadrat. A stainless-steel scoop was used to collect the upper 2 - 3 cm of beach sediment [2,3]. The sampled sediment samples were obtained from the high and drift waterlines at designated sites on the lagoon and beach locations [3]. Each piece of identified organic matter found in the quadrat was hand-selected and discarded. After sampling and separation, all the sediment samples collected were sealed in sterile Ziploc bags and placed in aluminium foil.

### 2.2 Methods

#### 2.2.1 Sample Treatment

The procedure described by [4] was modified to extract microplastics from sediment samples collected from the lagoon and beach waterlines. The sediment samples were air-dried and later sieved through 5 mm mesh stainless steel sieves. The plastic debris clinging to the 5 mm filter were carefully collected, and the sediment that passed through the sieve was rinsed with a saturated sodium chloride solution. The crude extracts from the density flotation procedure were strained through a 1 mm filter, and the residues were kept for further processing as described by [5], [6], [7]. Visual and stereomicroscopy identification of the MPs were performed, and the types of polymers were determined using an Agilent 630 Cary ATR-FTIR spectroscopy.

#### 2.2.2 Fourier Transform Infrared (FTIR) Analysis

The composition of all items identified as MPs was characterized following the procedure outlined by [3] using an Agilent Cary 630 FTIR spectrophotometer. Before each reading, the ATR plate was wiped off with alcohol. Each time a new set of measurements was completed, the surfaces were thoroughly cleaned before using the FTIR. For the identification of polymers, the Agilent polymer ATR library was employed. The following details pertain to the FTIR analysis: The ATR-FTIR system has an 8 cm^−1^ resolution, 32 sample scans, and a range of 4000–650 cm1. The absorption bands of each polymer were analysed and matched with the polymer ATR library with an acceptable match quality set at ≥80%. The results were confirmed using validated polymer spectral data [8].

#### 2.2.3 Sample Extraction for PAHs, OCPs, PCBs Analysis

MPs samples were placed in amber glass vials with 5 mL of cyclohexane/ethyl acetate mixture in 1:1 ratio. These were either hard or foam pieces, while composite samples were a mix of MP types. The vials were agitated for 2 min on a vortex machine and then placed in an ultrasonic bath for 20 min. The extracts were transferred to new vials, and the extraction procedures were repeated twice, with at least 15 mL aliquot recovered [4]. Prior to GC-Q-MS analysis, the extracts were concentrated to 1 mL, transferred to amber vials, and kept in the fridge at 4°C. The temperature of the column oven was programmed as follows: 100°C for 0 min, the increased at 20°C per minute to 180°C, then at 10°C per minute to 280°C, which was held for 2 min. The total run time was 16 min, followed by a 1 min post-run at 70°C.

#### 2.2.4 Quality Control

Procedural blanks were periodically measured using certified standards. Triplicate extractions were carried out in order to optimize the extraction efficiency. Extracts were homogenized and concentrated to 1 mL before GC-Q-MS analysis. The calibration curves' R^2^ values ranged from 0.995 to 0.998, 0.995 to 0.999, and 0.991 to 0.999 for PAHs, PCBs, and OCPs, respectively. All organic contaminants had spike recoveries between 81.3 and 127.3% [9]. Before the analysis, all equipment employed in the extraction procedure were washed, rinsed and dried with deionized water. To limit contamination by airborne fibres during sample preparation, extraction, and analysis, cotton-made laboratory coats, glassware and garments made from non-synthetic materials were used at all times.

## Ethics Statements

This study did not involve human or animal subjects, and no data from social media platforms were used.

## Funding

This research was supported by the Covenant University Centre for Research, Innovation and Discovery (CUCRID), through the Covenant University Seed Grant. The authors are grateful to Covenant University for providing publication assistance.

## Primary Data Availability

Microplastics and sorbed hydrophobic organic contaminants in lagoon and beach sediments (Raw Data) (Mendeley Data). The primary data as presented here represent the concentrations of polycyclic aromatic hydrocarbons (PAHs), polychlorinated biphenyls (PCBs) and organochlorine pesticides (OCPs) in sedimentary microplastic samples collected from coastal beaches and lagoonal ecosystem of the south east Atlantic Ocean, Gulf of Guinea, Nigeria. The aggregated concentrations of each organic pollutant were plotted as shown on tab labelled as Sum plot.

## CRediT authorship contribution statement

**Nsikak U. Benson:** Conceptualization, Visualization, Investigation, Data curation, Supervision, Project administration, Writing – original draft, Writing – review & editing. **Omowunmi H. Fred-Ahmadu:** Conceptualization, Methodology, Investigation, Data curation, Writing – review & editing. **Olusegun O. Ayejuyo:** Conceptualization, Investigation, Data curation, Supervision, Writing – review & editing.

## Declaration of Competing Interest

The authors declare that they have no known competing financial interests or personal relationships that could have appeared to influence the work reported in this paper.

## Data Availability

Microplastics and sorbed hydrophobic organic contaminants in lagoon and beach sediments (Original data) (Mendeley Data).

## References

[1] Benson N.U., Fred-Ahmadu O.H., Ayejuyo O. (2020). Microplastics and sorbed hydrophobic organic contaminants in lagoon and beach sediments. Mendeley Data.

[2] Karthik R., Robin R.S., Purvaja R., Ganguly D., Anandavelu I., Raghuraman R., Ramesh R. (2018). Microplastics along the beaches of southeast coast of India. Sci. Total Environ..

[3] Benson N.U., Fred-Ahmadu O.H. (2020). Occurrence and distribution of microplastics-associated phthalic acid esters (PAEs) in coastal psammitic sediments of tropical Atlantic Ocean, Gulf of Guinea. Sci. Tot. Environ..

[4] Camacho M., Herrera A., Gómez M., Acosta-Dacal A., Martínez I., Henríquez-Hernández L.A., Luzardo O.P. (2019). Organic pollutants in marine plastic debris from Canary Islands beaches. Sci. Tot. Environ..

[5] C.J. Masura, J. Baker, G. Foster, C. Arthur, Laboratory methods for the analysis of microplastics in the marine environment: recommendations for quantifying synthetic particles in waters and sediments, NOAA technical memorandum NOS-OR&R-48 (2015) 1–18. https://repository.library.noaa.gov.

[6] Fred-Ahmadu O.H., Ayejuyo O.O., Benson N.U. (2020). Dataset on microplastics and associated trace metals and phthalate esters in sandy beaches of tropical Atlantic ecosystems, Nigeria. Data Brief,.

[7] NOAA. (2015). Laboratory methods for the analysis of microplastics in the marine environment : recommendations for quantifying synthetic particles in waters and sediments. NOAA Technical Memorandum NOS-OR&R-48. (Issue July).

[8] Jung M.R., Horgen F.D., Orski S.V., Beers K.L., Balazs G.H., Lynch J.M. (2018). Validation of ATR FT-IR to identify polymers of plastic marine debris, including those ingested by marine organisms. Mar. Pollut. Bull..

[9] Fred-Ahmadu O.H., Tenebe I.T., Ayejuyo O.O., Benson N.U. (2022). Microplastics and associated organic pollutants in beach sediments from the Gulf of Guinea (SE Atlantic) coastal ecosystems. Chemosphere.

